# MOBILE INTERVENTION FOR FAMILY DEMENTIA CAREGIVERS: FROM FOCUS GROUPS TO SMARTPHONE PLATFORM

**DOI:** 10.1093/geroni/igac059.1229

**Published:** 2022-12-20

**Authors:** Felipe Jain, Saira Madarasmi, D J Ursal, Paulina Gutierrez-Ramirez, Abu Sikder, Sreya Banerjee, Liliana Ramirez Gomez

**Affiliations:** Depression Clinical Research Program / Massachusetts General Hospital / Harvard Medical School, Boston, Massachusetts, United States; Massachusetts General Hospital, Boston, Massachusetts, United States; Massachusetts General Hospital, Boston, Massachusetts, United States; Massachusetts General Hospital, Boston, Massachusetts, United States; Innovation Studio, Children's Hospital, Los Angeles, Los Angeles, California, United States; Massachusetts General Hospital, Boston, Massachusetts, United States; Massachusetts General Hospital, Harvard Medical School, Boston, Massachusetts, United States

## Abstract

There is growing recognition that in-person delivery of caregiver interventions limits scalability due to distance from locations where interventions are available, and costs associated with locating substitute care for the person living with dementia. Internet-based interventions are often not optimized for smartphones, which are more accessible than desktops to minoritized populations and those of lower socioeconomic status. In this study, focus groups were conducted with 17 English language and 12 Spanish language family dementia caregivers regarding needs for smartphone-based technological intervention. We employed an inductive and deductive driven mixed method analytic approach. Identified needs included psychoeducation regarding dementia, provision of caregiver skills information, activities for the person living with dementia, and relaxation techniques. Similarities and differences among preferences between the two populations were identified. Family dementia caregivers endorsed several needs for mobile intervention. The development of a new mobile application platform to meet these needs will be described.

